# Cost-Effectiveness of Bevacizumab Biosimilar LY01008 Combined With Chemotherapy as First-Line Treatment for Chinese Patients With Advanced or Recurrent Nonsquamous Non-Small Cell Lung Cancer

**DOI:** 10.3389/fphar.2022.832215

**Published:** 2022-04-19

**Authors:** Xia Luo, Qiao Liu, Zhen Zhou, Lidan Yi, Liubao Peng, Xiaomin Wan, Xiaohui Zeng, Chongqing Tan, Sini Li

**Affiliations:** ^1^ Department of Pharmacy, The Second Xiangya Hospital of Central South University, Changsha, China; ^2^ Menzies Institute for Medical Research, University of Tasmania, Hobart, TAS, Australia; ^3^ Department of Nuclear Medicine, PET Image Center, The Second Xiangya Hospital of Central South University, Changsha, China

**Keywords:** cost-effectiveness, NSCLC, bevacizumab, LY01008, biosimilar, China

## Abstract

**Objective:** To investigate whether LY01008, a locally developed bevacizumab biosimilar agent, is appropriate for widespread use among Chinese advanced or recurrent nonsquamous non-small cell lung cancer (NSCLC) patients, our current study was designed to evaluate the cost-effectiveness of first-line LY01008 combined with platinum-doublet chemotherapy versus chemotherapy alone from the perspective of the Chinese healthcare system.

**Material and Methods:** This economic evaluation designed a Markov model to compare the healthcare cost and quality-adjusted life-year (QALY) of first-line LY01008 combined with chemotherapy versus first-line chemotherapy. Transition probabilities, including disease progression, survival, and adverse event (AE)-related discontinuation of first-line treatment, were estimated using data from the clinical trials. Costs and health utilities were derived from local databases, hospitals, and published literature. Our base case analysis and scenario analysis focused on the cost-effectiveness of chemotherapy combined with a clinical trial dosage (15 mg/kg every 3-week cycle) and a real-world dosage (7.5 mg/kg every 3-week cycle) of LY01008, respectively.

**Results:** In the base case analysis, first-line LY01008 combined with chemotherapy was associated with an increase of 0.48 QALYs in effectiveness and an increase of CNY 189,988 (US$ 26,240) in healthcare costs compared with first-line chemotherapy, resulting an incremental cost-effectiveness ratio (ICER) of CNY 375,425 (US$ 54,430)/QALY. In the scenario analysis, first-line LY01008 combined with chemotherapy was associated with a mean healthcare cost of CNY 265,060 (US$ 38,429), resulting an ICER of CNY 221,579 (US$ 32,125/QALY) between first-line LY01008 combined with chemotherapy versus first-line chemotherapy. The parameters that determine the cost of LY01008 have the greatest impact on the cost-effectiveness results.

**Conclusion:** From the perspective of the Chinese healthcare system, first-line LY01008 at a real-world dosage combined with chemotherapy is likely to represent a cost-effective strategy compared with first-line chemotherapy alone for Chinese advanced or recurrent nonsquamous NSCLC patients.

## Introduction

Bevacizumab (Avastin, Genentech, Inc.) is the first approved monoclonal antibody directed against vascular endothelial growth factor (VEGF) that has been widely used in clinical practice to treat several malignancies such as non-small cell lung cancer (NSCLC) (Cohen et al., 2007; Zahn et al., 2019; Garcia et al., 2020). The phase III BEYOND trial of Chinese NSCLC patients has proven the efficacy of bevacizumab in prolonging overall survival (OS) and progression-free survival (PFS) when used in combination with carboplatin and paclitaxel as the first-line treatment for advanced or recurrent nonsquamous subtypes (Zhou et al., 2015). Based on this finding, the combination of bevacizumab with platinum-doublet chemotherapy was approved by the Chinese National Medical Products Administration (NMPA) in 2015 (Center for drug evaluation of National Medical Products Administration, 2015) and was also listed as a preferred first-line treatment option for this refractory disease in the latest Chinese Society of Clinical Oncology (CSCO) Guidelines (Guidelines Working Committee of Chinese society of Clinical Oncology, 2020).

Despite compelling clinical efficacy, the high cost of bevacizumab has largely limited its use and availability (Putrik et al., 2014; Baumgart et al., 2019). From the perspective of the Chinese healthcare system, two economic evaluations found that bevacizumab combined with chemotherapy was not cost-effective among patients with advanced nonsquamous NSCLC when compared with conventional chemotherapy (Zheng et al., 2018; Li et al., 2019). Since cancer is now a leading cause of premature death and has imposed a substantial economic burden on the national health system, the Chinese government has been engaged in sponsoring local scientists to develop bevacizumab biosimilar agents in recent years (Han et al., 2019; Yang et al., 2019; Shi et al., 2021). Bevacizumab biosimilars have shown similar benefits to the bevacizumab originator at a lower price, which could increase patients’ affordability and access to the treatment (National Medical Products Administration, 2019). Between December 2019 and June 2021, the Chinese NMPA have approved four domestic bevacizumab biosimilars used as the first-line treatment for advanced or recurrent nonsquamous NSCLC (National Medical Products Administration, 2019; National Medical Products Administration, 2021a; National Medical Products Administration, 2021b; National Medical Products Administration, 2021c). As reflected in the latest CSCO Guidelines for NSCLC treatment, bevacizumab biosimilars are recommended as an affordable alternative to the foreign bevacizumab (Guidelines Working Committee of Chinese society of Clinical Oncology, 2020).

LY01008 is a bevacizumab biosimilar developed by Boan Biotechnology Co., Ltd. (Yantai, Shandong, China). LY01008 in combination with platinum-based chemotherapy was approved for use based on the result of a phase III clinical trial that demonstrated the similar therapeutic benefit of LY01008 to the bevacizumab originator as first-line therapy in Chinese patients with advanced or recurrent nonsquamous NSCLC (Shi et al., 2021). The development of bevacizumab biosimilars has made a considerable impact on the Chinese healthcare system, given the huge beneficiary population. In terms of the incidence of lung cancer, China ranked first with 816,000 new cases recorded in 2020 globally, of which nearly half were diagnosed with advanced or recurrent NSCLC (Cao et al., 2021; Sung et al., 2021). Of the NSCLC patients, nearly three-quarters (approximately 300,000 cases) were classified as having a nonsquamous histologic type (Chen et al., 2016). To determine whether this locally developed bevacizumab biosimilar is appropriate for a widespread use among advanced or recurrent nonsquamous NSCLC patients, we conducted this study to evaluate the cost-effectiveness of the first-line LY01008 combined with platinum-doublet chemotherapy versus chemotherapy alone from the perspective of the Chinese health care system.

## Materials and Methods

### Overview

Through TreeAge Pro software (version 2021, https://www.treeage.com/) for Markov modeling and R software (version 4.0.4, http://www.r-project.org for survival fitting we conducted an indirect cost-effectiveness comparison between the first-line LY01008 combined with carboplatin/paclitaxel and the first-line carboplatin/paclitaxel alone among patients with advanced or recurrent nonsquamous NSCLC, from the perspective of the Chinese health care system. Efficacy and safety data of LY01008 combined with carboplatin/paclitaxel were derived from a recent clinical trial (ClinicalTrials.gov number: NCT03533127), and the data of carboplatin/paclitaxel alone were derived from the BEYOND trial (ClinicalTrials.gov number NCT01364012). This economic evaluation followed the Chinese guidelines for pharmacoeconomic evaluation (2020) (Chinese Pharmaceutical Association, 2020). Our study did not use individual patient-level data and was therefore deemed exempt from the approval of Chinese ethics review committees.

### Model Construction

**FIGURE 1:**
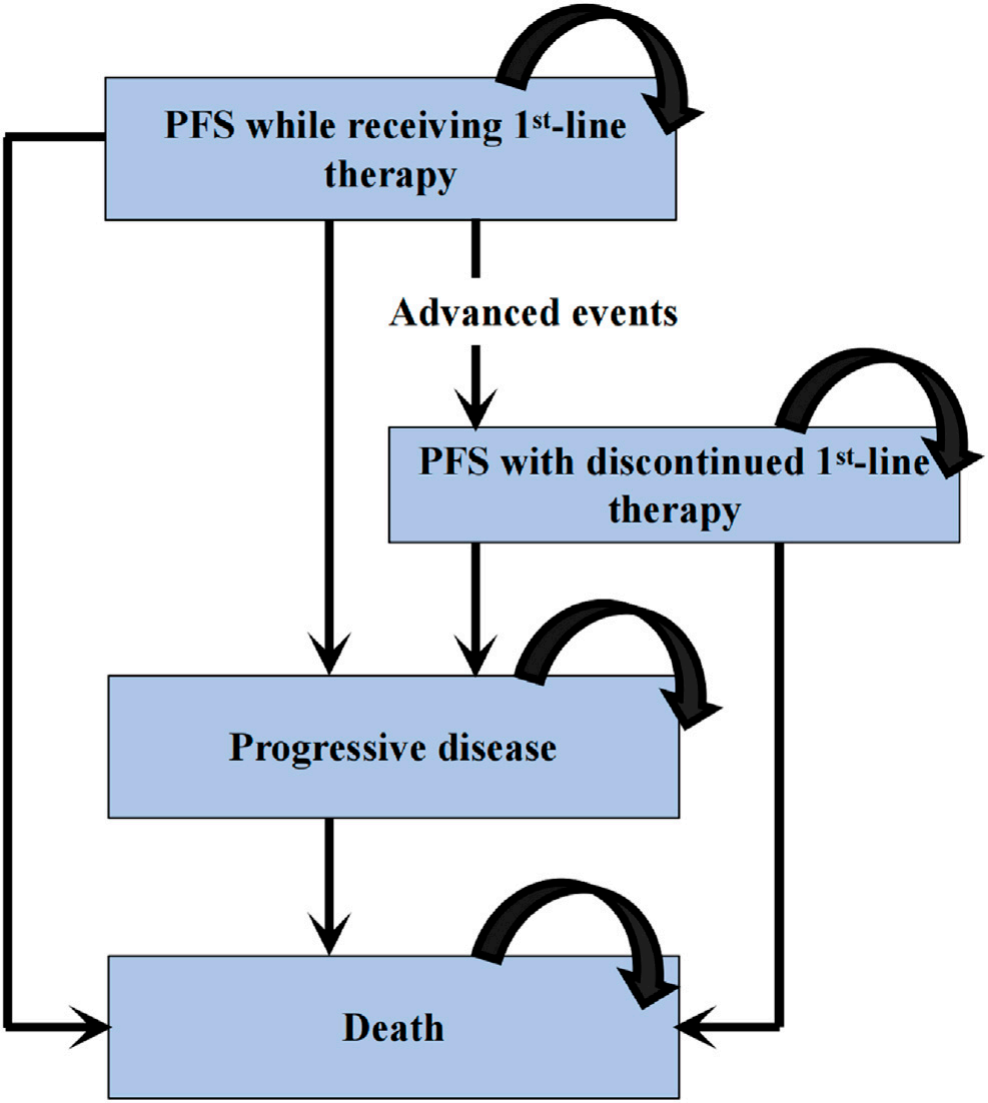
Diagram of the Markov Model. PFS, progression-free survival.

To simulate the progression of advanced or recurrent nonsquamous NSCLC, we built the Markov model composed of three main health states: progression-free survival (PFS), progressive disease (PD), and death. All model patients began from the PFS health state and were randomly assigned to two first-line treatments. According to the safety data reported in clinical trials, we considered that the model patients with the PFS health state may discontinue first-line therapies because of the occurrence of adverse events (AEs) (Zhou et al., 2015; Shi et al., 2021). Therefore, the PFS health state was divided into two substates: PFS health state while receiving first-line therapy and PFS health state with first-line therapy discontinued. Figure 1 illustrates the possible transitions between different health states. Patients with disease progression during the first-line treatment would enter the PD health state, in which a certain proportion of patients continue to receive subsequent anticancer therapy if they obtained sustained benefits, otherwise they were recommended to best supportive care (BSC) (Guidelines Working Committee of Chinese society of Clinical Oncology, 2020; Shi et al., 2021). To reflect the clinical practice in real-life scenarios, patients were proceeded to palliative care before death. Supplementary Table S1 provides detailed information regarding the first-line treatments used in this study.

The Markov model used a 3-week cycle and a 30-year time horizon to generate the incremental cost-effectiveness ratio (ICER) between the two competitive regimens that reflects the incremental healthcare cost consumed for each additional quality-adjusted life-year (QALY). We then compared the ICER with the willingness-to-pay (WTP) threshold defined as three times of China’s per capita GDP in 2020 [CNY 230,635 (US$ 33,438) per QALY) to assess whether the first-line LY01008 combined with chemotherapy is cost-effective (Chinese Pharmaceutical Association, 2020; National Bureau Of Statistics Of China, 2020). This study reported costs in both 2020 US$ and CNY, and costs and QALYs were discounted at an annual rate of 5% (Chinese Pharmaceutical Association, 2020).

### Transition Probabilities

Using the modeling approach described in our previous studies (Liu et al., 2021; Qiao et al., 2021) and the aggregated clinical trial data, we estimated transition probabilities between health states for the model patients assigned to the first-line LY01008 combined with chemotherapy and those assigned to chemotherapy alone. First, the PFS and OS of the chemotherapy group were extracted from the Kaplan–Meier (KM) curves published by the BEYOND trial investigators and best fitted and extrapolated by the Weibull distribution (Supplementary Figure S1). Second, for the chemotherapy group, the transition probability from the PFS health state to death at time *t* is calculated as: $S\left(t\right)=exp\left(-\lambda t^{\gamma }\right)$ ; the transition probability from the PFS health state to PD health state at a given cycle *t* is calculated as: $P\left(t\right)=1-exp\left[\left(\lambda {\left(t-1\right)}^{\gamma }\right)-\lambda t^{\gamma }\right]$ , where scale (λ) and shape (γ) were the Weibull parameters. Third, for the first-line LY01008 combined with chemotherapy group, the Weibull curves f were derived using the adjusted Weibull scale: $\left(\lambda_{LY01008+Chemotherapy}=\lambda_{Chemotherapy}\times \mathrm{HR}\right)$ and shape $\left(\gamma_{LY01008+Chemotherapy}=\gamma_{Chemotherapy}\right)$ , where the HRs of first-line LY01008 combined with chemotherapy relative to the first-line chemotherapy were generated by a network meta-analysis using R software due to the lack of clinical trials that compared two treatments head-to-head (Gu et al., 2019). Fourth, the transition probabilities between the two PFS substates were estimated based on the treatment discontinuations caused by AEs from each clinical trial (Zhou et al., 2015; Shi et al., 2021) (Supplementary Table S2 **)**.

### Costs and Effectiveness

All costs and utilities included in the model are outlined in Table 1. Drug prices were sourced from China’s health industry data platform (https://www.yaozh.com/) (China’s Health Industry Data Platform, 2021). For anthropometry-dependent dosage, we modeled the body weight of base case patients as 65 kg, the body surface area as 1.72 m^2^, and the creatinine clearance rate as 70 ml/min (Lu et al., 2017; Wan et al., 2019). In calculating the cost of treating AEs, we used the safety data reported for the bevacizumab–chemotherapy group in the BEYOND trial to model the LY01008 combined with chemotherapy group, given the similar safety profile between LY01008 and bevacizumab (Avastin, Genentech, Inc.) (Shi et al., 2021). The AE cost of each first-line treatment was calculated by multiplying the frequency of AEs by the corresponding treatment cost estimated using data from local hospitals, and then the sum value was obtained by combining these products. The cost of general management for NSCLC, such as routine follow-up, subsequent anticancer therapy, BSC, and palliative care, was derived from the previous literature (Qiao et al., 2021).

**TABLE 1:** Model inputs.

Variable	Baseline value	Range	Distribution	Source
Survival				
Weibull survival model for first-line chemotherapy				
OS	Scale = 0.004716; shape = 1.533854	Fixed in DSA	Fixed in PSA	Estimated^a^
PFS	Scale = 0.003867; shape = 2.335407	Fixed in DSA	Fixed in PSA	Estimated^a^
HRs for first-line LY01008–chemotherapy vs. chemotherapy				
OS	0.654	0.302–1.410	Normal	Estimated^b^
PFS	0.405	0.078–2.160	Normal	Estimated^b^
1-cycle probability of treatment discontinuation due to AEs				
First-line LY01008 combined with chemotherapy	0.004298	0.002149–0.006448	Beta	Estimated^c^
First-line chemotherapy	0.006372	0.003186–0.009558	Beta	Estimated^c^
Costs (US$)				
LY01008 price per 15 mg	24.99	12.49–37.48	Gamma	Local charge
Paclitaxel price per 175 mg	57.51	28.75–86.26	Gamma	Local charge
Carboplatin price per 6 mg	1.37	0.69–2.06	Gamma	Local charge
Nivolumab price per 4.5 mg	60.35	30.18–90.53	Gamma	Local charge
Routine follow-up cost per cycle	55.60	27.80–83.40	Gamma	[24]
Subsequent therapy cost per cycle	854.05	427.02–1,281.08	Gamma	[24]
BSC cost per cycle	337.50	168.75–506.25	Gamma	[24]
Palliative care cost per cycle	2,627.80	1,313.90–3,941.70	Gamma	[24]
AEs cost for LY01008 combined with chemotherapy	1,025.82	512.91–1,538.73	Gamma	Estimated^d^
AEs cost for chemotherapy	745.01	372.51–1,117.52	Gamma	Estimated^d^
Utilities				
PFS health state	0.856	0.718–0.994	Beta	[29]
PD health state	0.768	0.595–0.941	Beta	[29]
Disutility for LY01008 combined with chemotherapy	0.061	0.031–0.092	Beta	Estimated^d^
Disutility for chemotherapy	0.080	0.040–0.119	Beta	Estimated^d^
Others				
Discount rate (%)	5	0–8	Fixed in PSA	[22]
Patient weight (kg)	65	32.5–97.5	Normal	[27]
Body surface area (m^2^)	1.72	0.86–2.58	Normal	[27]
Creatinine clearance rate	70	35–105	Normal	[28]

OS, overall survival; PFS, progression-free survival; HR, hazard ratio; BSC, best supportive care; AEs: adverse events; PD, progressive disease.

a Estimated by the survival fitting implemented in R software.

b Estimated by the network meta-analysis implemented in R software.

c Estimated in Supplementary Table S2.

d Estimated in Supplementary Table S3.

Treatment effectiveness was measured in QALY, which is calculated as a health utilities-weighted life expectancy (overall survival). Chinese-specific health state utility data of advanced NSCLC were used in the model (Shen et al., 2018). The disutilities caused by grade III/IV AEs during first-line treatment were also considered in the model (Nafees et al., 2017) and calculated as a frequency-weighted average. The costs and disutilities associated with AEs are detailed in Supplementary Table S3.

### Statistical Analysis

Sensitivity analyses were performed to test the robustness of the cost-effectiveness model. First, we conducted deterministic sensitivity analysis (DSA) to assess the impact of the variation of each individual parameter on the main cost-effectiveness result. We made utilities and HRs varied across their respective 95% Cis, discount rate varied between 0 and 8% (Chinese Pharmaceutical Association, 2020), and other parameters varied within ±50% of their baseline values. Second, we performed probabilistic sensitivity analyses (PSA) to assess the impact of variations of multiple parameters on the model outputs. During PSA, we used the appropriate distribution to describe each parameter and randomly sampled the model parameters from the corresponding distribution for 1,000 Monte Carlo simulations. The ranges and distributions regarding model parameters are outlined in Table 1.

We also performed a scenario analysis in which the patients assigned to the first-line LY01008 combined with chemotherapy group were administered LY01008 of 7.5 mg/kg every 3-week cycle (half of the clinical trial dosage), the dosage generally used in real-life clinical practice in China.

## Results

### Incremental Cost-Effectiveness Ratios

In our base case analysis, first-line LY01008 combined with chemotherapy was associated with an increased effectiveness of 0.48 QALYs and an increased healthcare cost of CNY 180,988 (US$ 26,240) compared with first-line chemotherapy. These increases yielded an ICER of CNY 375,425 (US$ 54,430)/QALY for first-line LY01008 combined with chemotherapy versus first-line chemotherapy (Table 2). In the scenario analysis, treating patients with 7.5 mg/kg LY01008 reduced the mean healthcare cost of first-line LY01008 combined with chemotherapy to CNY 265,060 (US$ 38,429). Therefore, the ICER between first-line LY01008 combined with chemotherapy and first-line chemotherapy was estimated to be CNY 221,579 (US$ 32,125)/QALY (Table 2).

**TABLE 2:** Summary of simulation results.

Analysis	Incremental				ICER, $/QALY
	Cost, $	QALYs	Cost, $	QALYs	
Base case analysis					
First-line chemotherapy	22,943	1.63			
First-line LY01008 combined with chemotherapy	49,182	2.11	26,240	0.48	54,430
Scenario analysis					
First-line chemotherapy	22,943	1.63			
First-line LY01008 combined with chemotherapy	38,429	2.11	15,487	0.48	32,125

QALYs, quality-adjusted life-years; ICER, incremental cost-effectiveness ratio.

### Sensitivity Analysis

**FIGURE 2:**
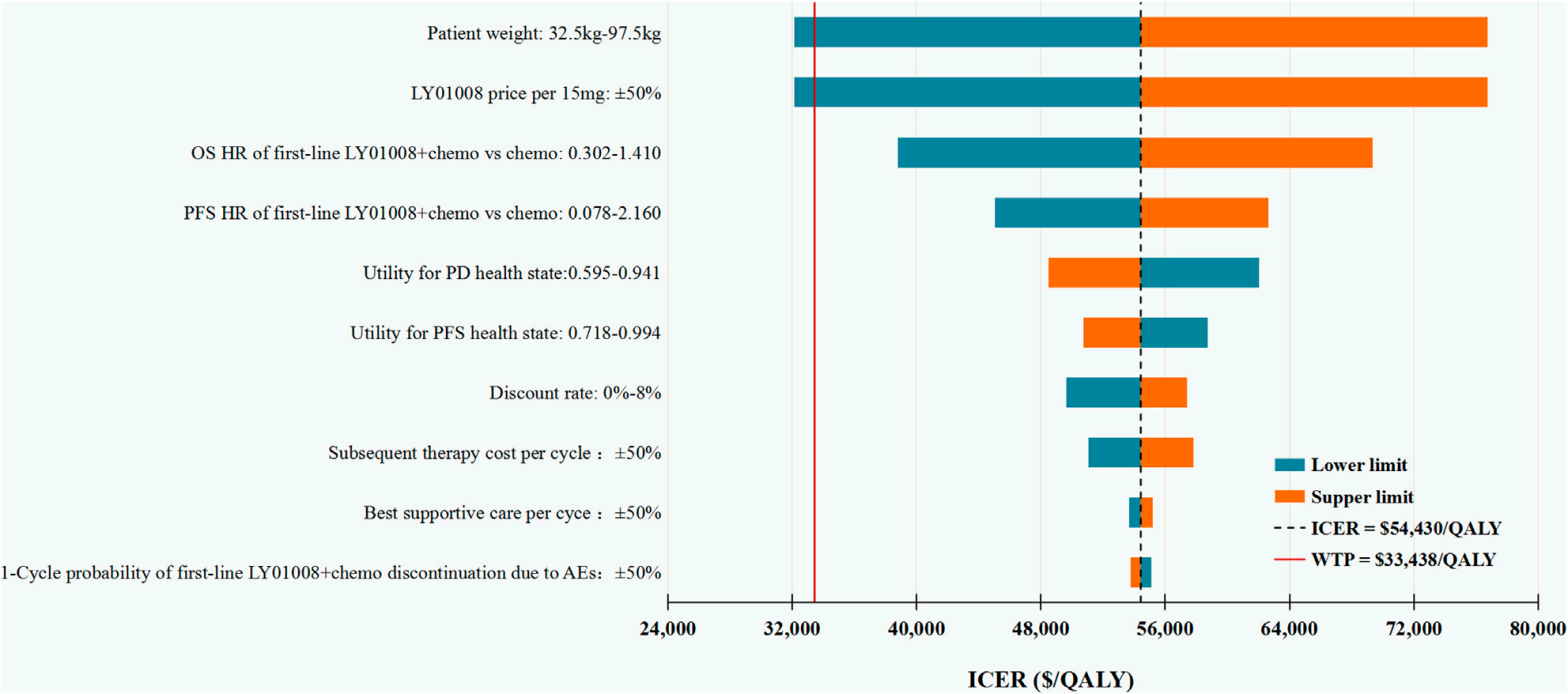
Deterministic sensitivity analysis for the base case analysis. ICER, incremental cost-effectiveness ratio; QALYs, quality-adjusted life-years; OS, overall survival; PFS, progression-free survival; HR, hazard ratio; PD, progressive disease; BSC, best supportive care; AEs: adverse events.

The results of DSA suggested that under the WTP threshold of CNY 230,635 (US$ 33,438)/QALY, first-line LY01008 combined with chemotherapy was cost-effective only at the lower limits of patient weight and LY01008 price per 15 mg, with estimated ICERs of CNY 221,572 (US$ 32,124)/QALY and CNY 221,510 (US$ 32,115)/QALY, respectively. Other model parameters only changed our results moderately, with the resulting ICERs surpassing the WTP threshold. The top 10 parameters associated with the ICER are shown in Figure 2.

The PSA revealed that when assuming a WTP threshold of CNY 230,635 (US$ 33,438/QALY), the cost-effectiveness probability of first-line LY01008 combined with chemotherapy compared with first-line chemotherapy was 23.5%, and the probability was expected to increase with the increasing WTP thresholds (Supplementary Figure S2).

## Discussion

In this economic evaluation among Chinese advanced or recurrent nonsquamous NSCLC patients, we found that when compared with the classic first-line carboplatin/paclitaxel chemotherapy, adding LY01008 at a clinical trial dosage (15 mg/kg every 3-week cycle) to this chemotherapy was not cost-effective, whereas adding a real-world dosage of LY01008 (7.5 mg/kg every 3-week cycle) to this chemotherapy was likely to represent a cost-effective treatment option. Our sensitivity analysis results revealed that only patient weight and LY01008 price per 15 mg, the parameters that determine the cost of LY01008, were able to change the model results.

This study raises several concerns regarding the impact of drug cost on the selection of cancer treatment. This is particularly the case in China, where more than one-third of the newly diagnosed lung cancer cases in the world came from nonsquamous NSCLC (Cao et al., 2021). It can be expected that even a small rise in the drug cost can considerably increase the economic burden imposed on the Chinese healthcare system (WHO, 2012). Confronted with such challenges, the Chinese government has decreed a series of policies to reduce the market price of anticancer drugs in recent years, including supporting the local research on self-developed cancer drugs (Li et al., 2021) and running national drug price negotiations with suppliers (Tang et al., 2020). Since 2016, the Chinese government has been implementing several rounds of drug price negotiations with pharmaceutical manufacturers, resulting in the price of many expensive anticancer drugs reduced by more than 50% (Ministry of Human Resources and Social Security of the People’s Republic of China, 2018). LY01008 evaluated in this study is a locally developed bevacizumab biosimilar. Its low market price may contribute to its unique advantage over the originator products in the drug price negotiation. Therefore, the price of LY01008 is likely to be reduced in the near future. In this context, we can expect that the cost-effectiveness of first-line LY01008 combined with chemotherapy in Chinese advanced or recurrent nonsquamous NSCLC patients will be improved.

Cost reduction in biosimilars is beneficial from many aspects. First of all, the lower market price of biosimilars will force the pharmaceutical manufacturers to reduce the drug price of originator products so as to stave off the wide use of biosimilars and on the other hand, promote the research and development of next-generation biologics (Moorkens et al., 2020). Second, cost saving in biosimilars such as LY01008 will improve the patients’ affordability and accessibility to the cancer treatment and extend their life, thus reducing the disease burden of cancer in China (Dutta et al., 2020). Third, the medical expenditure saved through using cheaper biosimilars can be reallocated to subsidize and reimburse treatments for other diseases, such as cardiovascular diseases, so as to promote the benign and sustainable development of the whole health care system.

Following the LY01008 cost-related parameters, HRs of first-line LY01008 combined with chemotherapy versus first-line chemotherapy had the greatest impact on the cost-effectiveness result in our study, underscoring the need for data from a head-to-head clinical trial to validate our model. In this analysis, although the HRs used to estimate transition probabilities were generated by a network meta-analysis, the DSA results were not significantly altered by the uncertainty in HRs, suggesting that the use of more accurate data is unlikely to change our conclusions. Furthermore, in our scenario analysis using a real-world dosage of LY01008 (7.5 mg/kg every 3-week cycle), we found that the use of first-line LY01008 combined with chemotherapy is likely a cost-effective therapeutic option. This dosage is widely used in China for the following two reasons: first, a previous clinical trial observed similar clinical efficacy between chemotherapy in combination with 15 mg/kg versus 7.5 mg/kg of bevacizumab among Asian patients with advanced or recurrent nonsquamous NSCLC (Mok et al., 2011); second, China’s huge population base makes lower dose of bevacizumab, thus lower cost, being more affordable to Chinese patients.

To our knowledge, this is the first cost-effectiveness analysis of locally developed bevacizumab biosimilar focusing on Chinese advanced or recurrent nonsquamous NSCLC patients. This study has great implications for the Chinese government to balance its enormous population and limited healthcare resources. With almost the entire population participating in the national health insurance, the Chinese government becomes the largest payer of medical services and healthcare. The Chinese government has thus faced an unprecedented challenge to provide affordable cancer treatments for Chinese patients. Though the Chinese government has been engaged in sponsoring the development of local biosimilars coupled with national drug price negotiation, more effort is needed on improving patients’ access to the innovative cancer treatments.

This study has several limitations. First, although we accurately simulated the disease process of patients based on the clinical efficacy and safety data derived from large clinical trials, the posttrial outcomes for patients were uncertain. Second, we used the safety data reported for the bevacizumab–chemotherapy group in the BEYOND trial to model the LY01008–chemotherapy group in the current study because of the high similarity in the safety profile between participants receiving LY01008 and bevacizumab (Avastin, Genentech, Inc.) (Shi et al., 2021). Third, potential biases existed in using some non-native resources to estimate costs and utilities. However, our cost-effectiveness results were not particularly sensitive to these nonlocal model inputs. Fourth, our economic evaluation compared the first-line LY01008 combined with chemotherapy with first-line chemotherapy indirectly; more robust cost-effectiveness analyses are needed when head-to-head clinical trial data are available.

In conclusion, first-line LY01008 at a real-world dosage of 7.5 mg/kg every 3-week cycle combined with chemotherapy is likely to represent a cost-effective strategy compared with first-line chemotherapy alone for Chinese patients with advanced or recurrent nonsquamous NSCLC.

## Data Availability

The original contributions presented in the study are included in the article/Supplementary Material, further inquiries can be directed to the corresponding author.
