# Supplementary figures and images for: Cost-Effectiveness of Bevacizumab Biosimilar LY01008 Combined With Chemotherapy as First-Line Treatment for Chinese Patients With Advanced or Recurrent Nonsquamous Non-Small Cell Lung Cancer
Source: Front Pharmacol. 2022 Apr 19;13:832215. doi: 10.3389/fphar.2022.832215 (PMC9062292; doi:10.3389/fphar.2022.832215)

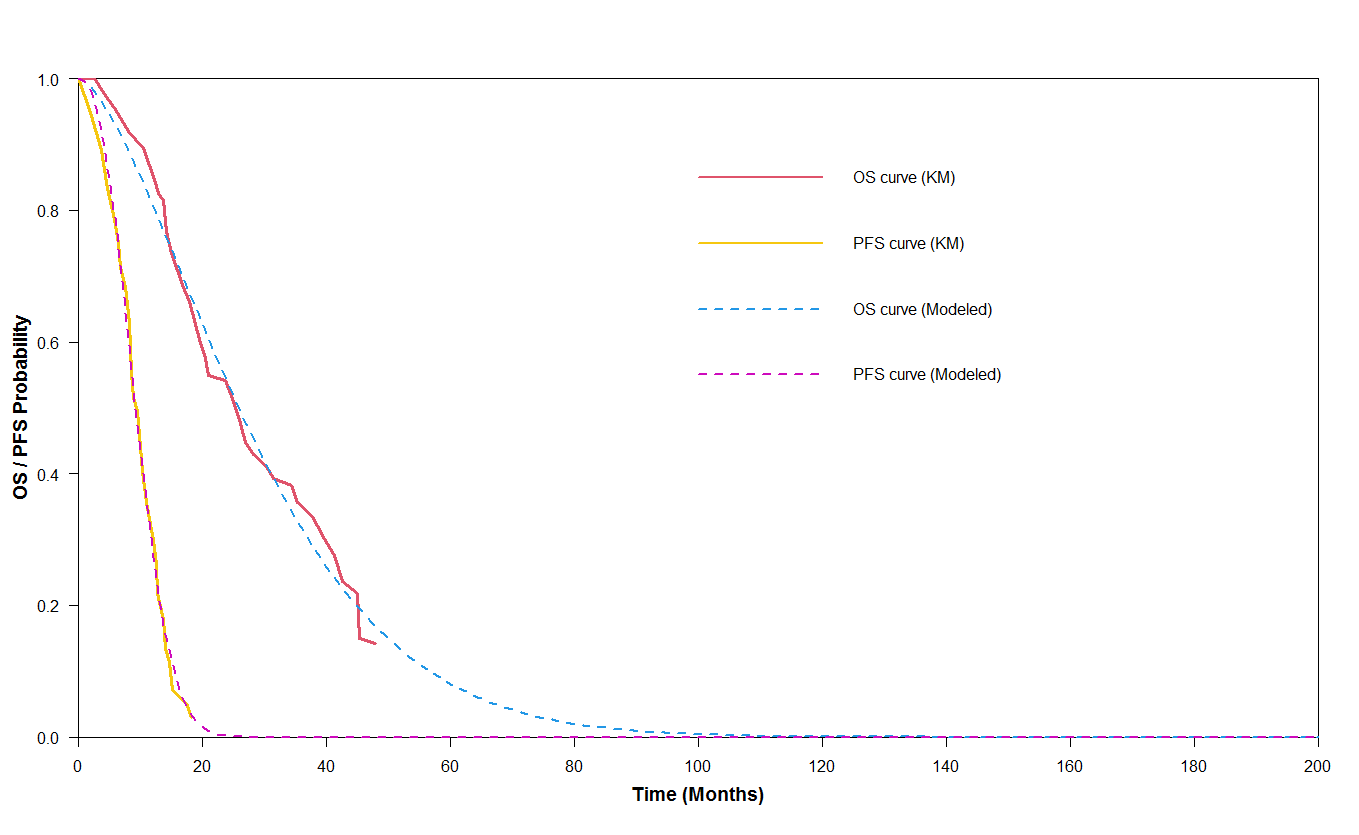

Supplement: Supplementary file 1 [file Image1.TIFF]

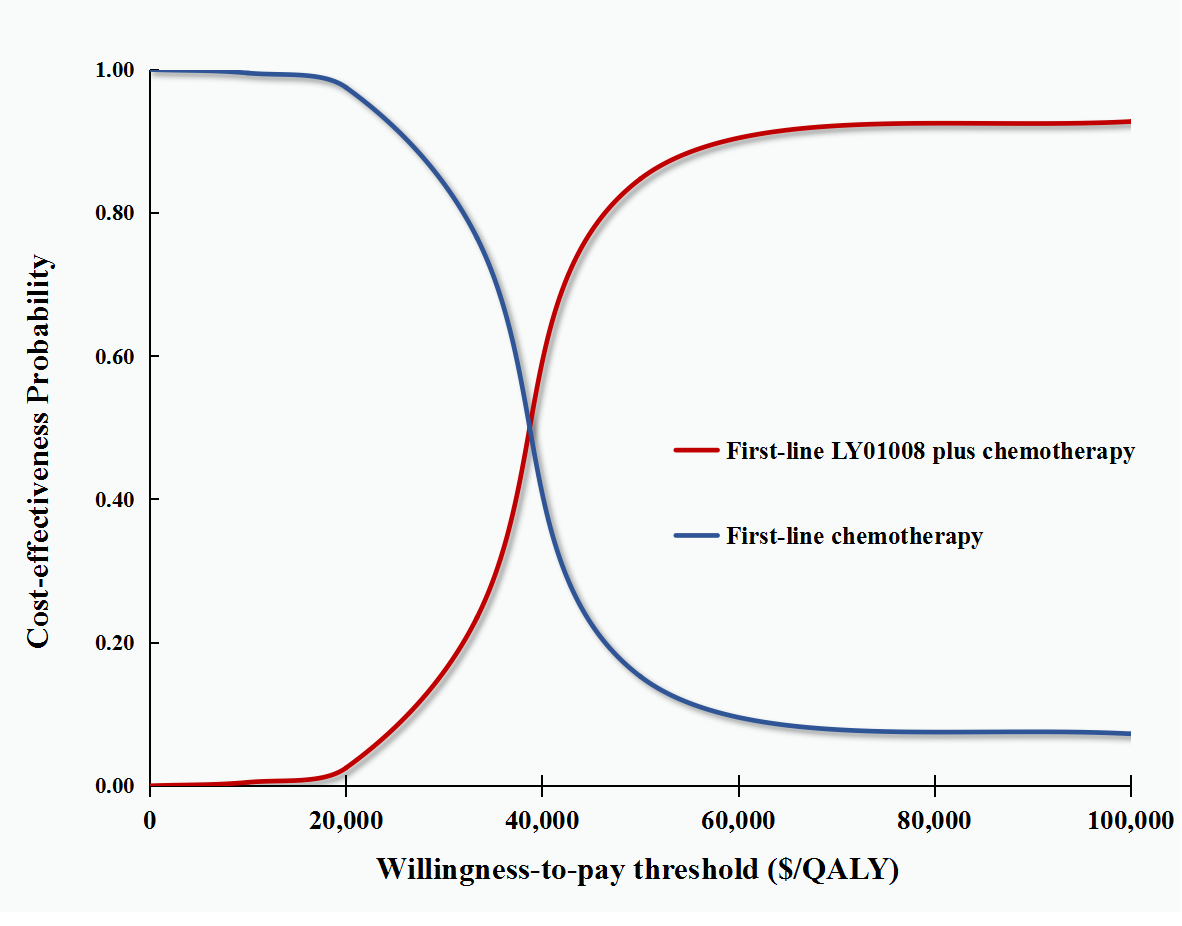

Supplement: Supplementary file 3 [file Image2.tif]
