# Supplementary material for: Cost-Effectiveness of Bevacizumab Biosimilar LY01008 Combined With Chemotherapy as First-Line Treatment for Chinese Patients With Advanced or Recurrent Nonsquamous Non-Small Cell Lung Cancer
Source: Front Pharmacol. 2022 Apr 19;13:832215. doi: 10.3389/fphar.2022.832215 (PMC9062292; doi:10.3389/fphar.2022.832215)
Supplement: Supplementary file 2 [file Table1.DOCX]

Table S1. First-line and second-line treatments in the model.

| **Regimens** | **Dose Size** | **Treatment schedule** | **Proportion of subsequent anticancer therapy** |
| --- | --- | --- | --- |
| First-line  LY01008 plus chemotherapy | LY01008, 15mg/kg | On day 1 of each 3-week cycle | 70%^a^ |
|  | Carboplatin, AUC 6.0mg/ml/min | On day 1 of each 3-week cycle for up to six cycles |  |
|  | Paclitaxel, 175mg/m^2^ | On day 1 of each 3-week cycle for up to six cycles |  |
| First-line  chemotherapy | Carboplatin, AUC 6.0mg/ml/min | On day 1 of each 3-week cycle for up to six cycles | 71% |
|  | Paclitaxel, 175mg/m^2^ | On day 1 of each 3-week cycle for up to six cycles |  |

*AUC, area under the curve.*

*^a^Since information on subsequent therapy related to LY01008 combined chemotherapy were not published in the clinical trial, we modeled the proportion of patients receiving subsequent therapy in the LY01008 combined chemotherapy was identical to that of bevacizumab-chemotherapy group in the BEYOND trial.*
