# Supplementary material for: Cost-Effectiveness of Bevacizumab Biosimilar LY01008 Combined With Chemotherapy as First-Line Treatment for Chinese Patients With Advanced or Recurrent Nonsquamous Non-Small Cell Lung Cancer
Source: Front Pharmacol. 2022 Apr 19;13:832215. doi: 10.3389/fphar.2022.832215 (PMC9062292; doi:10.3389/fphar.2022.832215)
Supplement: Supplementary file 4 [file Table2.DOCX]

Table S2. Proportion and Probability of first-line treatment discontinuation due to AEs.

| **Regimen** | **Number of patients**  **(probability)** | **Median OS**  **(months)** | **Instantaneous rate** | **1-cyle probability** |
| --- | --- | --- | --- | --- |
| First-line LY01008 plus chemotherapy  (N=323) | 45 (0.139319 ) | 24.38 | 0.004308 | 0.004298 |
| First-line chemotherapy  (N=134) | 20 (0.149254 ) | 17.70 | 0.006393 | 0.006372 |

*OS: overall survival; AEs: adverse events.*

*The following formula was applied to convert the probabilities of AEs-related treatment discontinuation during a clinical trial period into a 1-cylce probability of the events:* $P=1-exp(-rt)$*, where p indicates the probability, r is the instantaneous rate and t is the time period.*
