# Supplementary material for: Cost-Effectiveness of Bevacizumab Biosimilar LY01008 Combined With Chemotherapy as First-Line Treatment for Chinese Patients With Advanced or Recurrent Nonsquamous Non-Small Cell Lung Cancer
Source: Front Pharmacol. 2022 Apr 19;13:832215. doi: 10.3389/fphar.2022.832215 (PMC9062292; doi:10.3389/fphar.2022.832215)
Supplement: Supplementary file 5 [file Table3.docx]

Table S3. Proportion, costs and disutility of grade III/IV AEs considered in the model.

| AEs | Proportion (%) | | Cost per event($)^a^ | Disutility |
| --- | --- | --- | --- | --- |
|  | First-line LY01008 plus carboplatin/paclitaxel | First-line carboplatin/paclitaxel |  |  |
| Neutropenia | 23% | 28% | 588.22 | 0.20 |
| Anaemia | 7% | 11% | 2011.03 | /^c^ |
| Thrombocytopenia | 7% | 9% | 1324.05 | /^c^ |
| Bone marrow failure | 11% | 3% | 5057.44 | /^c^ |
| Febrile neutropenia | 3% | 5% | 1466.63 | 0.42 |
| Hypertension | 5% | 1% | 1110.92 | 0.04 |
| Diarrhea | 1% | 3% | 120.97 | 0.07 |
| Proteinuria | 4% | 0% | /^b^ | /^c^ |
| Back pain | 0% | 2% | 6.91 | /^c^ |
| Estimated AEs Costs and disutility | | |  |  |
| AEs cost for first-line LY01008 plus carboplatin/paclitaxel, $ | | | 1025.82 |  |
| AEs cost for first-line carboplatin/paclitaxel, $ | | | 745.01 |  |
| AEs disutility for first-line LY01008 plus carboplatin/paclitaxe | | |  | 0.061 |
| AEs disutility for first-line carboplatin/paclitaxel | | |  | 0.080 |

*AEs, adverse events.*

*^a^The AEs treatment cost used in this analysis were estimated from local hospitals.*

*^b^Based on the local oncologists’ opinions and clinical practices, patients with severe proteinuria would be discontinued from LY01008 treatment or reduced the dosage.*

^c^*The disutility regarding these AEs were not reported.*
